# Evaluating the methodological quality of coordinate-based meta-analyses: the qual-CBMA checklist

**DOI:** 10.3389/fnimg.2026.1753543

**Published:** 2026-05-13

**Authors:** Joaquim Radua, Lydia Fortea, Anton Albajes-Eizagirre, Claudia Eickhoff, Miquel A. Fullana, Paolo Fusar-Poli, David Mataix-Cols, Jae Il Shin, Aleix Solanes, Masoud Tahmasian, Eduard Vieta, Enric Vilajosana, Simon B. Eickhoff, Veronika I. Müller

**Affiliations:** 1 Institut d'Investigacions Biomèdiques August Pi i Sunyer (IDIBAPS), Barcelona, Spain; 2 Universitat de Barcelona, Institut de Neurociències, Barcelona, Spain; 3 Mental Health Research Networking Center (CIBERSAM), Madrid, Spain; 4 Institute of Clinical Neuroscience and Medical Psychology, Medical Faculty and University Hospital Düsseldorf, Heinrich Heine University, Düsseldorf, Germany; 5 Hospital Clinic de Barcelona, Clinical Institute of Neuroscience, Barcelona, Spain; 6 Department of Brain and Behavioral Sciences, University of Pavia, Pavia, Italy; 7 OASIS Service, South London and Maudsley NHS Foundation Trust, London, United Kingdom; 8 Department of Psychosis Studies, Institute of Psychiatry, Psychology & Neuroscience, King's College London, London, United Kingdom; 9 Centre for Psychiatric Research, Department of Clinical Neuroscience, Karolinska Institutet, Stockholm, Sweden; 10 Department of Pediatrics, Yonsei University College of Medicine, Seoul, Republic of Korea; 11 Research Centre Jülich, Institute of Neuroscience and Medicine (INM-7), Jülich, Germany; 12 Institute of Systems Neuroscience, Medical Faculty and University Hospital Düsseldorf, Heinrich Heine University, Düsseldorf, Germany; 13 Department of Nuclear Medicine, Faculty of Medicine and University Hospital Cologne, University of Cologne, Cologne, Germany

**Keywords:** activation likelihood estimation, checklist, meta-analysis, neuroimaging, seed-based d mapping

## Abstract

Voxel-based meta-analyses—also known as coordinate-based meta-analyses (CBMAs)—are powerful tools for synthesizing evidence from neuroimaging studies in human neuroscience, including investigations of psychological functions and differences in brain disorders. To achieve their full potential in accurately assessing the evidence, CBMAs should adhere to established best-practicmpe guidelines, such as the “Ten Simple Rules” published in 2018. Yet, even when studies report following these recommendations, the degree to which individual items are applicable or fully addressed is often unclear. To better support the evaluation of methodological rigor—which the 10 rules already promote but are not always consistently applied—, the developers of the most used CBMA methods followed a Delphi-style iterative process to create a reporting checklist focused on the methodological quality of CBMAs (Qual-CBMA). Qual-CBMA comprises criteria (e.g., preregistration, systematic search, homogeneous study characteristics, etc.) that authors should verify and comment on explicitly in the checklist (and, when unmet, also in the manuscript). The checklist encourages rigor and transparency by prompting authors to identify potential methodological limitations and to discuss their relevance—or irrelevance—in the context of their specific study. The checklist is designed as an aid to make reporting clearer and more transparent, not as a tool for evaluating whether authors have done something incorrectly. In this context, a high-quality CBMA is not defined by meeting every criterion, but by clearly commenting on the criteria—and explaining when unmet criteria are appropriately not applicable given the study’s objectives. We encourage authors to submit the Qual-CBMA checklist, together with their accompanying comments, when publishing new CBMAs, thereby reinforcing transparency and rigorous methodology and advancing understanding in cognitive neuroscience and clinical conditions.

## 1 Introduction

Over the last decades, neuroimaging methods have become increasingly popular across disciplines such as basic and applied human neuroscience. As a result, thousands of studies using techniques such as magnetic resonance imaging (MRI) and positron emission tomography (PET) have investigated the neural correlates of psychological functions and brain disorders. To integrate these findings, several research groups have developed voxel-based meta-analytic approaches—commonly referred to as coordinate-based meta-analyses (CBMA). Such syntheses are particularly valuable given that low replicability has been observed even when reanalyzing the same dataset using different analytical strategies (Botvinik-Nezer et al., 2020). Thus, neuroimaging meta-analytic approaches can identify neural patterns that are consistently found across studies.

As a relatively young field, CBMAs initially lacked clear, public methodological guidelines. In this regard, it is important to note that general reporting checklists, such as PRISMA (Page et al., 2021; Radua, 2021), offer only limited applicability to CBMAs because of their specific methodological characteristics. For example, they do not consider key CBMA procedures, such as transforming all data into a common space, ensuring consistency in the brain coverages of the included datasets, or ensuring that the statistical thresholds do not vary across brain regions (e.g., as in small volume corrections). Conversely, general reporting checklists require information that cannot be commonly provided in a CBMA, such as an effect size estimate for each study.

This changed only a few years ago, when a set of best-practice recommendations for CBMA were published (Müller et al., 2018; Tahmasian et al., 2019). These so-called “ten simple rules” emphasize the importance of defining a specific research question, considering the power of the meta-analysis, preplanning and preregistering the study, collecting and organizing data systematically, ensuring comparable coverage and reference spaces, double-checking data, balancing sensitivity and susceptibility to false positives, adjusting for multiple contrasts, assessing potential biases and robustness, and reporting transparently. Since then, many CBMAs have claimed to adhere to these best-practice guidelines, in which we had included an initial checklist.

However, a recent study found citation to these recommendations is inconsistent, with nearly 8% of quotations containing errors such as misinterpretation, oversimplification, or unsupported claims (Yeung, 2023). Such errors are not infrequent in neuroimaging and evidence synthesis more broadly. For example, a recent analysis showed that a widely cited report promoting uncorrected statistical thresholds in neuroimaging was often invoked to justify their use, despite the fact that most citing studies deviated substantially from the recommended parameters (Yeung, 2024). Similarly, another analysis found that a commentary critical of a scale used to assess the quality of observational studies in systematic reviews was frequently cited as supporting the scale, even though it explicitly argued the opposite (Stang et al., 2018).

In addition, we conducted an exploratory search for published CBMAs, which suggested heterogeneous reporting of how specific recommendations were addressed (Supplementary file). Potential reasons why adherence to the guidelines has been inconsistently reported may be that the checklist was optional and, when used, several items required detailed open-ended responses in the Supplementary file without corresponding comments or justifications in the checklist or the manuscript. Furthermore, for some rules, the criteria for determining compliance were unclear.

It is important to emphasize that inconsistent reporting does not imply that the underlying work was conducted incorrectly. However, such inconsistencies can be confusing for readers. In some cases, authors may state that they followed the recommendations but did so only partially. In other cases, authors may need to acknowledge that certain recommendations could not be met (for example, regarding the minimum number of included studies), even though the work itself is methodologically sound and scientifically valuable when considered in context (e.g., a first-ever meta-analysis of a rare condition).

To address these problems and to provide researchers with a tool that helps them to reflect and report the concordance of meta-analytical studies with the guidelines, we developed a “PRISMA-like” checklist of criteria—Quality of CBMA (Qual-CBMA)—focused on the methodological quality of CBMAs according to the best-practice guidelines. We adopted a Delphi-style iterative refinement process: an initial proposal was co-created by two authors (JR and SBE), after which two structured rounds of feedback from all authors were conducted to converge on the final draft and criteria. We aimed to create a tool that supports authors by making expectations clearer, rather than evaluating their methodological decisions. Importantly, the checklist is not intended to impose rigid requirements that are incompatible with the adaptive and iterative nature of CBMAs, but to promote transparent reporting of how methodological decisions were made.

The Qual-CBMA serves a dual purpose: it provides guidance for authors to design and report their CBMA transparently, and it offers a structured framework for readers. The checklist is intended as a supportive guide to help authors communicate methodological decisions transparently, not as an evaluation of whether their work is ‘correct’ or ‘incorrect.’ It is not intended to generate numeric scores or rankings, but rather to facilitate transparent and contextualized reporting of methodological strengths and limitations. We hope that its use will encourage authors of future CBMAs to enhance the methodological rigor of their work and, consequently, strengthen the evidence such studies provide.

Finally, it is also important to note that the Qual-CBMA uses clear binary items to increase clarity and accountability, and the requirement to comment on every response both discourages superficial compliance and allows authors to justify legitimate deviations. Importantly, a CBMA can still be methodologically robust even if several items are marked negatively. What matters is that these deviations are transparently commented.

## 2 Methods

The Qual-CBMA checklist includes 15 specific criteria (described below), each of which should be verified and commented upon in the checklist, and if not met, also in the manuscript. The presence of 15 criteria corresponding to the original 10 rules reflects a deliberate effort to improve clarity: several rules were operationalized by dividing them into multiple, more concrete criteria. First, the rule “be specific about your research question” was split into three criteria addressing (i) imaging modalities or measures, (ii) functional paradigms, and (iii) conditions or disorders. Second, the rule “collect and organize your data” was divided into two criteria, one concerning the use of search keywords across multiple scientific databases and the other requiring clearly specified inclusion and exclusion criteria. Third, the rule “ensure that all included experiments use the same search coverage and identify and adjust differences in reference space” was subdivided into three criteria: (i) whether statistical thresholds are independent of brain region, (ii) whether brain coverage is consistent across studies, and (iii) whether coordinates are converted to a common reference space. Fourth, the rule “be transparent in reporting” was split into two criteria, one focusing on the description of datasets, contrasts, and samples, and the other assessing whether data and code required to reproduce the CBMA are publicly available or sufficient information is reported to allow replication. Finally, we did not operationalize the rule “find a balance between sensitivity and susceptibility to false positives”; this issue is instead addressed separately in the discussion of statistical significance (see below).

Rather than producing an overall score, the checklist is intended to ensure that readers are aware of any potential methodological limitations while allowing authors to place these limitations in context—or to demonstrate that, in their specific CBMA, they do not constitute actual limitations. That said, methodological quality complements but does not replace quantitative evidence strength, and both dimensions should be considered when interpreting the robustness of meta-analytic results. Importantly, the criteria are not meant to prescribe what authors must do, but to prompt explicit reporting so that readers can better understand the methodological context. A criterion marked as ‘No’ is not a negative judgment—it simply indicates that additional context will help readers understand why the item does or does not apply to the study. Many well-conducted CBMAs will naturally mark several items as ‘No,’ depending on their aims and available data.

### 2.1 Qual-CBMA criterion 1—preregistration (corresponds to the rule 7)

This criterion assesses whether the CBMA protocol was preregistered in a public database, such as the International Prospective Register of Systematic Reviews (PROSPERO)^1^ or the Open Science Framework (OSF),^2^ or published in a peer-reviewed journal (e.g., Fortea et al., 2021), which is now mandatory for most meta-analyses. Preregistration of methodology can increase transparency and credibility by clarifying which aspects of the study were planned in advance and which decisions were made adaptively in response to the retrieved literature. In addition, preregistration of the aim and hypothesis helps prevent duplicated efforts, as it allows researchers to verify whether another group is already conducting a similar CBMA before initiating a new one. However, we explicitly acknowledge that CBMAs often require iterative refinement of search strategies, inclusion criteria, and analytic decisions based on the number, heterogeneity, and characteristics of the available studies. As a result, preregistration is not a mandatory requirement for a high-quality CBMA, and deviations from an initial plan should be expected rather than viewed as problematic. The purpose of this criterion is therefore not to make preregistration mandatory, but to encourage authors to clarify whether a protocol existed, how it guided the final analyses, and which decisions were made *post hoc*. When preregistration was not performed, authors should briefly comment on this and, where relevant, explain how analytic flexibility was handled transparently.

### 2.2 Qual-CBMA criteria 2–3—systematic search (corresponds to rule 3)

These criteria evaluate whether the authors conducted a systematic literature search, defined as using predefined keywords in multiple scientific databases and applying explicit inclusion and exclusion criteria. A systematic search ensures that the CBMA is based on a comprehensive and unbiased set of studies and increases transparency and reproducibility–especially if accompanied by the list of screened studies.

### 2.3 Qual-CBMA criteria 4–6—specific modalities, measures, paradigms, conditions, and disorders (correspond to rule 1)

These three criteria ensure that the included imaging modalities (e.g., structural MRI, DTI, fMRI), measures (e.g., cortical thickness, gray matter VBM, BOLD response), functional paradigms (e.g., n-back, emotional faces), or conditions (e.g., psychological distress) and disorders (e.g., schizophrenia) are clearly defined. Importantly, these criteria apply to each analysis within a study. For example, if authors first perform separate CBMAs for different MRI modalities and subsequently assess the overlap across modalities, the criteria should be evaluated on the separate CBMAs rather than on the multimodal conjunction/overlap analysis.

To provide practical guidance, we propose that imaging modalities or measures be considered the same when any differences among them are primarily methodological, and the datasets could, in principle, be reanalyzed using a single software pipeline. For example, studies employing different VBM implementations (e.g., SPM-DARTEL vs. FSL-VBM) or parameters (e.g., modulated vs. unmodulated images) would be regarded as using the same modality or measure, because these differences are mainly methodological and all datasets could be reprocessed using a single VBM pipeline. We acknowledge that modulated and unmodulated VBM capture related but not identical properties of gray matter, reflecting volume and density, respectively. Nonetheless, in the context of a CBMA, these differences can reasonably be regarded as primarily methodological.

Regarding functional paradigms, we propose that they be considered the same when they examine the same underlying psychological function and are therefore expected to produce similar activation maps. This would apply, for instance, to different tasks assessing working memory.

Regarding conditions or disorders, we propose that they be considered the same when they target the same population—for example, patients diagnosed with a specific disorder.

Conversely, authors should explicitly comment when modalities, measures, paradigms, conditions, or disorders are only similar rather than the same. For instance, a CBMA investigating anatomical or volumetric differences between groups might combine voxel-based morphometry (VBM) and surface-based morphometry (SBM, e.g., FreeSurfer). Although these approaches are related, they differ conceptually: VBM assesses regional volume, whereas SBM may measure cortical thickness. Reanalyzing VBM datasets using SBM software would thus entail a conceptual change (the studies assessed gray matter volume, but the findings are reinterpreted as cortical thickness). Similarly, a CBMA could mix task-based fMRI and PET studies—techniques that are methodologically related but not equivalent, as their datasets cannot be analyzed with the same pipeline. Finally, a CBMA could combine functional paradigms probing related yet distinct psychological functions (e.g., emotion regulation and attention), or disorders (e.g., patients with different anxiety disorders).

Authors may legitimately argue that specific criteria do not apply to their research questions. For example, some studies may aim to identify brain regions that are altered in a specific disorder regardless of the MRI modality and, therefore, justify combining different modalities as long as the rationale and interpretation remain consistent with that aim.

### 2.4 Qual-CBMA criterion 7—adjustment for multiple GLM contrasts (corresponds to rule 5)

This criterion examines whether the CBMA included only one GLM contrast per dataset or, alternatively, adequately adjusted for multiple contrasts derived from overlapping samples (see, e.g., Tahmasian et al., 2019; Turkeltaub et al., 2012; Alegria et al., 2016). If such an adjustment was applied, the approach should be fully justified and described or appropriately referenced.

### 2.5 Qual-CBMA criterion 8—studies used space-uniform statistical thresholds (corresponds to part of rule 4)

This criterion assesses whether each individual study applied a uniform statistical threshold across all brain regions and voxels (while the threshold can perfectly differ among studies). For example, if a study uses a permissive threshold (e.g., uncorrected *p* < 0.01) within a region of interest (ROI) but a more conservative threshold (e.g., family-wise error rate (FWER) < 0.05) to the rest of the brain, the CBMA should only include those results that meet the conservative threshold, and the results of the more liberal threshold should be excluded, whether in the ROI or in the rest of the brain. This space-nonuniform statistical threshold may happen, for instance, with small-volume corrections (SVC) and hidden ROIs.

This rule prevents biased results toward specific overrepresented regions of interest and ensures that the assumption that each voxel has *a priori* the same chance of being activated is not violated. It is important to note that this criterion does not require all studies included in the CBMA to have used the same statistical threshold; instead, it requires that each individual study applied a consistent threshold across the whole brain.

### 2.6 Qual-CBMA criteria 9–10—same brain or tissue coverage and space (corresponds to part of rule 4)

These criteria ensure that the authors conducted their analyses within the same brain or tissue coverage and spatial reference framework. For example, in an analysis of cortical thickness, both the individual studies and the CBMA itself must be restricted to the cortex, and the data from the different studies reporting coordinates in different stereotactic spaces must be transformed into a common stereotactic space (e.g., MNI).

### 2.7 Qual-CBMA criterion 11—double extraction (corresponds to rule 6)

This criterion evaluates whether the data extraction was conducted independently by at least two researchers and afterward checked. This practice reduces the likelihood of human error in data collection, coding, and analysis, thereby increasing the reliability of the CBMA results. Ideally, CBMAs should report the agreement between the raters, as recommended in behavioral meta-analyses (Johnson and Hennessy, 2019).

### 2.8 Qual-CBMA criterion 12—amount of data (corresponds to rule 2)

The twelfth criterion relates to the number of datasets included in a CBMA and, consequently, to its overall statistical power. We acknowledge that power estimation should ideally consider both the number of datasets and the expected effect size. However, the latter cannot be easily estimated for most CBMA methods. Moreover, as most CBMA approaches rely on the convergence of findings across studies, the number of included independent experiments is important beyond the overall number of participants in these experiments.

To meet the twelfth criterion, a CBMA should include at least 17 independent peak-coordinate datasets—though 23 might be needed for structural MRI. These thresholds are based on prior simulation work showing that a standard ALE meta-analysis requires around 17 datasets to achieve adequate power (Eickhoff et al., 2016; Frahm et al., 2022; Frahm et al., 2023). In addition, these numbers moderately approximate the optimal information size (OIS) recommended by the GRADE criteria for intervention meta-analyses (Guyatt et al., 2011). The OIS concept states that the total sample size in a meta-analysis should be at least as large as the sample size required for an adequately powered single study. For CBMAs with 17 moderately sized datasets (approximately 20 individuals for one-sample studies or 33 individuals per group for two-sample studies; see below for further details), this corresponds to an overall sample size of roughly 340 participants for one-sample studies or 561 participants per group for two-sample studies—approximately sufficient to detect one-sided small effects (*d* = 0.2) with 80% power at *p* = 0.001.

That said, if the criterion is not met but the studies are large or statistical maps are available, we suggest that authors provide a comment on this. To help motivate such a comment, we offer a heuristic adjustment of the count of studies according to their sample sizes and the availability of statistical maps. As detailed below, in this adjustment, each study is weighted according to its sample size (Table 1), and studies providing statistical maps count twice. Importantly, this sample-size adjustment refers to how studies are counted when estimating the adjusted number of studies *a priori*, and not to the internal weighting of coordinates or contrasts within the meta-analytic algorithms, which already account for sample size in their statistical models.

**Table 1:** Adjustment for the sample size of included experiments when counting the number of datasets.

A. Count of one-sample studies		B. Count of two-sample studies			
Sample size	Study count	Sample size per group	Study count	Sample size per group	Study count
5	0.04	5	0.03	39	1.10
6	0.07	6	0.05	40	1.12
7	0.11	7	0.07	41	1.13
8	0.16	8	0.10	42	1.14
9	0.23	9	0.13	43	1.15
10	0.30	10	0.16	44	1.16
11	0.37	11	0.19	45	1.17
12	0.45	12	0.23	46	1.18
13	0.53	13	0.27	47	1.19
14	0.61	14	0.31	48	1.19
15	0.69	15	0.35	49	1.2
16	0.76	16	0.39	50	1.2
17	0.83	17	0.44	51	1.21
18	0.89	18	0.48	52	1.21
19	0.94	19	0.52	53	1.22
20	0.99	20	0.56	54	1.22
21	1.03	21	0.6	55	1.22
22	1.07	22	0.64	56	1.23
23	1.10	23	0.68	57	1.23
24	1.13	24	0.72	58	1.23
25	1.15	25	0.76	59	1.23
26	1.17	26	0.79	60	1.24
27	1.18	27	0.83	61	1.24
28	1.20	28	0.86	62	1.24
29	1.21	29	0.89	63	1.24
30	1.22	30	0.92	64	1.24
31	1.22	31	0.94	65	1.24
32	1.23	32	0.97	66	1.24
33	1.23	33	0.99	67	1.24
34	1.24	34	1.01	68	1.24
35	1.24	35	1.03	≥69	1.25
36	1.24	36	1.05		
37	1.24	37	1.07		
≥38	1.25	38	1.09		

On the one hand, even if a CBMA includes fewer than 17 (or 23 for VBM) independent datasets, it may still be adequately powered if the number of participants in the individual studies is huge. Conversely, a CBMA including 20 studies may still be underpowered if each study only includes ~10 participants. To account for this, we propose that in the adjusted number of datasets, studies with small sample sizes count as less than one dataset, while studies with large sample sizes count as slightly more than one dataset. Specifically, each study would be counted as the ratio between its statistical power to detect a one-sided very large effect (*d* = 1) with *p* = 0.001 and a conventional target power of 80%. A one-sample study with 20 participants yields approximately 80% power. It would count as one dataset, whereas a study with 12–13 participants yields roughly 40% power and would count as 0.5 datasets. A study with 38 or more participants yields close to 100% power and would count as 1.25 datasets. Similarly, a two-sample study with 33–34 participants per group yields about 80% power and thus would count as one dataset, a study with 18–19 participants per group yields approximately 40% power and would count as 0.5 datasets, and a study with 69 or more participants per group yields about 100% power and would count as 1.25 datasets. This formula heavily penalizes tiny studies while avoiding excessive weighting of large ones, as no study counts for more than 1.25 datasets. For convenience, the corresponding adjustment values are provided in Table 1 (and its R code in the Supplementary file).

On the other hand, statistical maps convey much richer information than peak coordinates. To quantify this difference, we conducted simulations of univariate meta-analyses comprising 10–20 one-sample studies, varying the number of studies that simulated providing statistical maps (i.e., available *t*-values regardless of significance) with the remaining studies simulating only providing peak information (i.e., t-values reported only if *p* < 0.001). We used MetaNSUE to estimate the meta-analytic *z*-values accounting for unreported non-statistically significant effects (Radua et al., 2015; Albajes-Eizagirre et al., 2019). Specifically, we simulated 100 meta-analyses for each combination of (a) total number of studies, (b) number of studies simulating statistical maps, and (c) effect size (*d* = 0.2, 0.5, or 0.8), and then averaged the resulting *z*-statistics for each combination. For meta-analyses with no studies simulating statistical maps, we fitted simple models of the square root of the number of studies as a function of the mean z-statistic, separately for each effect size. Using these models, we estimated the equivalent number of studies for all meta-analyses according to the mean *z*-statistics. Finally, we fitted interceptless regressions modeling the difference between the equivalent and the actual total number of studies as a function of the number of studies simulating statistical maps. These analyses indicated that each study providing a statistical map contributed about four times more information than a peak-coordinate study. However, this factor strongly depended on effect size (ranging from about 10 for *d* = 0.2 to 1.15 for *d* = 0.8). To adopt a conservative and straightforward correction, we therefore propose that in the adjusted number of studies, each study providing a statistical map be counted as only two datasets of that sample size—e.g., a map based on only five participants would only count as 2 × 0.04 = 0.08 datasets. Again, we acknowledge that this counting may not generalize, but this scaling is only a pragmatic approximation.

For simplicity, this criterion can be evaluated using the unadjusted number of datasets alone. The proposed weighting scheme is optional and intended only as an aid for contextualizing the amount of available evidence when authors consider it informative.

If this criterion is not met, authors may provide context—for example, explaining that their research question addresses a narrower or emerging area where fewer studies exist. A small number of datasets is not inherently problematic; this criterion helps readers understand the scope of the available evidence.

### 2.9 Qual-CBMA criterion 13—diagnostics (corresponds to rule 9)

This criterion encourages the use of diagnostic analyses, such as assessing potential publication bias, which has been shown to affect neuroimaging research (David et al., 2013; Fusar-Poli et al., 2014; David et al., 2018) as well as measures of robustness or number of contributing datasets to convergence. To meet this criterion, the CBMA should evaluate potential publication bias, robustness, and/or convergence contributions using an appropriate method or tool (see, e.g., Radua et al., 2014; Acar et al., 2018; Tamon et al., 2024; Reimann et al., 2025; Müller et al., 2025). We acknowledge that such diagnostics may not be feasible in CBMAs that include very few studies; as with the other criteria, authors may comment on this.

### 2.10 Qual-CBMA criteria 14–15—transparent reporting (corresponds to rule 10)

The fourteenth criterion concerns the transparent description of the datasets, GLM contrasts, and samples included in the CBMA—for example, specifying relevant sample and experimental characteristics such as specific contrast included and the mean age and percentage of female participants.

The fifteenth criterion represents a further step toward open science: reporting should be sufficiently detailed to allow replication. In other words, the data and code used in the CBMA should be available through a public repository or website, enabling independent researchers to retrieve them and reproduce the analyses. If data cannot be shared because of good reason (which should be justified), every information needed for a replication must be reported in detail, e.g., the source of the coordinates of each dataset has to be specified—for example (Müller et al., 2017). Manuscripts stating only that “data are available upon reasonable request” would not meet this criterion.

Finally, when using publicly available software such as ALE or SDM, it is sufficient for authors to specify the software version and the exact analyses conducted.

### 2.11 A note on the statistical significance

While we do not argue that smaller *p*-values might indicate stronger evidence, we deliberately decided that the Qual-CBMA checklist should not consider the statistical significance of findings. Including such a criterion could inadvertently encourage p-hacking—that is, researchers trying multiple CBMA methods or varying analytical parameters until achieving statistically significant results.

Instead, we recommend that the scientific community become accustomed to and accept large-scale CBMAs that yield no statistically significant findings after correction for multiple comparisons. In the absence of corrected findings, CBMAs may report null results and, as an add-on, report uncorrected findings. The main argument against reporting uncorrected findings (beyond corrected findings) is that they may increase the risk of false positives, ultimately reducing reproducibility. However, there are also arguments for presenting results across a range of statistical thresholds—from more conservative to more liberal—, which can help overcome the limitations of a binary “significant vs. non-significant” framework (Belbasis et al., 2015; Radua et al., 2018; Fullana et al., 2020). Indeed, in a large-scale reproducibility study (Botvinik-Nezer et al., 2020), differences between analytical approaches were less pronounced when results were compared across multiple thresholds rather than at a single cut-off.

We anticipate that some readers may consider it “unfair” that a CBMA that includes, for instance, 14 studies with highly significant findings (e.g., FWER < 0.01) must still acknowledge having fewer than 20 datasets. However, compelling evidence requires a substantial amount of data. In studies comparing patients and controls, limited data may lead to random but significant differences in confounding variables, which can partly drive neuroimaging results. When the amount of data is large, the influence of such random confounders becomes negligible, as sample characteristics converge toward the population mean and thus become more comparable across groups.

### 2.12 Do Qual-CBMA criteria evaluate the quality or strength of the evidence?

The Qual-CBMA criteria do not directly assess the strength of evidence; instead, they evaluate methodological quality, including the adequacy of the data. Nevertheless, methodological quality is a fundamental pillar of evidence grading systems in other fields (Alonso-Coello et al., 2016; Fusar-Poli and Radua, 2018; Radua and Koutsouleris, 2024). Indeed, the traditional pyramid of evidence primarily considers the type of study design and does not even account for the amount of data available.

### 2.13 How to use the Qual-CBMA checklist

Researchers should include the checklist provided in Table 2 (available at https://imardgroup.com/qual-cbma/) and explicitly comment on all criteria and especially those that are not met. For example, if a CBMA meets all criteria except for combining unrelated imaging modalities because this was intentioned, we recommend that the authors make this clear, e.g.: “Please note that the only reason our CBMA did not meet all criteria is that we intentionally combined unrelated imaging modalities, as derived from one of the aims of our study, even if we had enough studies to conduct a separate CBMA for each modality”. In this example, should the study have fewer than 17 studies per modality, authors should be transparent and acknowledge it, e.g., “Please note that a reason our CBMA did not meet all criteria is that we combined unrelated imaging modalities, as derived from one of the aims of our study, plus the fact that we had not enough studies to conduct a separate CBMA for each modality.”

**Table 2:** Methodological quality of coordinate-based meta-analyses (Qual-CBMA) checklist.

Criterion:		Check:
1. The protocol of the CBMA is preregistered in a publicly available database.		☐ Yes ☐ No
*Comment here, and if unmet, also in the manuscript:*		
2. Studies are searched using search keywords in multiple scientific databases.		☐ Yes ☐ No
*Comment here, and if unmet, also in the manuscript:*		
Inclusion/exclusion criteria	3. The CBMA specifies clear inclusion/exclusion criteria.	☐ Yes ☐ No
	*Comment here, and if unmet, also in the manuscript:*	
	4. Studies use the same imaging modality (e.g., structural MRI) and measure (e.g., gray matter VBM).	☐ Yes ☐ No
	*Comment here, and if unmet, also in the manuscript:*	
	5. If applicable, same functional paradigm (e.g., n-back). [criterion not applicable to modalities with no functional paradigms]	☐ Not applicable ☐ Yes ☐ No
	*Comment here, and if unmet, also in the manuscript:*	
	6. If applicable, same condition (e.g., psychological distress) or disorder (e.g., only schizophrenia). [criterion not applicable to studies not investigating conditions or disorders]	☐ Not applicable ☐ Yes ☐ No
	*Comment here, and if unmet, also in the manuscript:*	
	7. Only one GLM contrast per dataset or multiple contrasts are adequately adjusted, and the approach is fully justified or referenced.	☐ Yes ☐ No
	*Comment here, and if unmet, also in the manuscript:*	
	8. Only results in which the statistical threshold does not depend on the brain region (i.e., excluding results obtained using region-liberal thresholds, such as from small-volume corrections or other “hidden ROIs” unless they meet the whole-brain threshold).	☐ Yes ☐ No
	*Comment here, and if unmet, also in the manuscript:*	
9. The brain or tissue coverages of the included datasets and the CBMA agree (e.g., all studies and the CBMA cover the gray but not the white matter, even if a few studies fail to cover small parts of the gray matter).		☐ Yes ☐ No
*Comment here, and if unmet, also in the manuscript:*		
10. Data are transformed into a common space (e.g., MNI) before conducting the analyses (automatic step with software such as ALE or SDM).		☐ Yes ☐ No
*Comment here, and if unmet, also in the manuscript:*		
11. Data are collected independently by at least two investigators and then checked.		☐ Yes ☐ No
*Comment here, and if unmet, also in the manuscript:*		
12. Total number of independent datasets is at least 17. Note: As an optional contextualization, authors may weight studies according to sample size (Table 1) and count studies providing statistical maps as two datasets of that sample size.		☐ Yes ☐ No
*Comment here, and if unmet, also in the manuscript:*		
13. Assessment of potential publication bias, robustness, and/or experiment contribution with an appropriate method (e.g., Egger tests, Galbraith plots, number of additional noise studies, number of contributing experiments)		☐ Yes ☐ No
*Comment here, and if unmet, also in the manuscript:*		
Transparent reporting	14. Description of datasets, contrasts, and samples (e.g., specific contrast, mean age, and percentage of females in the samples).	☐ Yes ☐ No
	*Comment here, and if unmet, also in the manuscript:*	
	15. Data and code required to reproduce the CBMA are publicly available, or sufficient information is reported to allow replication.	☐ Yes ☐ No
	*Comment here, and if unmet, also in the manuscript:*	

ALE: activation likelihood estimation; CBMA: coordinate-based meta-analysis. GLM: general linear model. MRI: magnetic resonance imaging; PET: positron emission tomography; ROI: region of interest. SBM: surface-based morphometry. SDM: seed-based d-mapping (previously signed differential mapping); VBM: voxel-based morphometry. Qual-CBMA checklist does not consider the statistical significance of the findings. Instead, it recommends that all the agents get used to and accept CBMAs with no findings after correction for multiple comparisons.

Table 3 provides an example case use.

**Table 3:** Example case use.

Criterion	Response	Example comment
1. Preregistration	☑ No	The CBMA was not preregistered because the protocol was created retrospectively during a broader systematic review.
2. Systematic search	☑ Yes	The databases and keywords are provided in the Methods.
3. Clear inclusion/exclusion criteria	☑ Yes	The inclusion/exclusion criteria are provided in the Methods.
4. Same imaging modality & measure	☑ Yes	All studies used BOLD fMRI task activation.
5. Same functional paradigm	☑ Yes	All studies used explicit emotion reappraisal tasks.
6. Same condition/disorder	☑ Yes	All studies were on healthy adults.
7. One contrast per dataset or adjustment	☑ Yes	We included one contrast per dataset
8. Space-uniform thresholding	☑ Yes	ROI/SVC peaks were only included if surpassing whole-brain thresholds.
9. Same brain or tissue coverage	☑ No	Two studies had partial coverage (omitting cerebellum) and were excluded in sensitivity analyses.
10. Common reference space	☑ Yes	All coordinates were transformed to MNI.
11. Double extraction	☑ No	Data was extracted by one investigator.
12. ≥17 datasets (or adjusted number)	☑ Yes	We included 18 datasets (19.4 after sample-size weighting).
13. Diagnostics conducted	☑ Yes	Funnel plots are provided in Figure 3.
14. Dataset description transparency	☑ Yes	Dataset description is provided in Table 1.
15. Reproducible reporting	☑ Yes	Data sources are referenced and scripts are available on OSF.

How the comments could be written in the manuscript: “We applied the Qual-CBMA checklist to evaluate methodological transparency and alignment with best-practice recommendations. Three criteria were not met: (1) the study was not preregistered because the protocol was developed retrospectively within a larger systematic review; (2) two included studies had slightly reduced brain coverage (i.e., cerebellum not fully acquired), and these were excluded in sensitivity analyses; and (3) data extraction was performed by a single investigator without independent duplication.”

A manuscript may also include multimodal CBMAs, for instance, combining a CBMA of regional homogeneity (ReHo) analyses of resting-state fMRI data with a CBMA of fractional anisotropy (FA) analyses of diffusion-weighted imaging (DWI) data. In such cases, we recommend applying the Qual-CBMA criteria separately to each modality, but not to the combined multimodal analysis. In another common scenario, a manuscript may include a main CBMA encompassing all studies together, as well as subgroup analyses, for example, one CBMA for all anxiety disorders and separate CBMAs for individual anxiety disorders. In these cases, the Qual-CBMA criteria should be applied independently to each CBMA.

### 2.14 Availability of data

We include the R code for the sample size simulations in the Supplementary file.

## 3 Discussion

Despite CBMAs claiming to follow the best-practice guidelines (Müller et al., 2018; Tahmasian et al., 2019), recent research has found citation to these guidelines contain misinterpretations, oversimplifications, or unsupported claims (Yeung, 2023), and our exploratory search suggested that certain aspects—such as protocol preregistration, diagnostic tests, or double checking—were inconsistently reported—likely reflect differences in reporting practices rather than methodological shortcomings. To help overcome these issues, we developed a checklist to assess the methodological quality of CBMAs—the Qual-CBMA checklist (Table 2). The Qual-CBMA provides a structured framework comprising 15 specific criteria derived from the best-practice “simple rules” (Müller et al., 2018).

The checklist can be applied to each individual CBMA within a multi-CBMA study—such as separate analyses for different modalities or diagnostic groups—and should ideally be included as a Supplementary Table. By encouraging consistency, transparency, and critical reflection on potential limitations, the Qual-CBMA aims to enhance the reliability and interpretability of neuroimaging meta-analyses and to facilitate reproducibility and cumulative progress in the field.

Importantly, the Qual-CBMA criteria do not take into account the statistical significance of the findings—a deliberate decision to avoid encouraging p-hacking. Instead, the checklist includes a note recommending that researchers, reviewers, and editors become accustomed to and accept CBMAs that yield no findings after correction for multiple comparisons. In other words, the acceptance of a manuscript should not depend on the presence of statistically significant results. That said, we acknowledge that researchers may differ on whether, in the absence of corrected findings, a CBMA should report no results or report uncorrected findings. On this point, we did not reach full agreement.

The Qual-CBMA checklist is intentionally designed as a simple and broadly applicable tool, and this design choice entails several limitations. First, the use of binary criteria and fixed thresholds inevitably creates artificial distinctions (e.g., a CBMA based on 16 datasets is unlikely to be meaningfully different from one based on 17). Similarly, in research areas where effect sizes are huge, even small studies may already detect them consistently, making these thresholds less informative. Second, the checklist is largely algorithm-agnostic and therefore cannot reflect all method-specific assumptions or optimal practices for individual CBMA approaches. Third, some criteria may not be applicable to all research questions or study designs, requiring authors to provide contextual justification rather than strict compliance. These limitations reflect a trade-off between simplicity, clarity, and methodological nuance. Future iterations of the Qual-CBMA could address these issues by incorporating optional graded or continuous criteria, algorithm-specific extensions, or weighted approaches, while preserving the checklist’s primary goal of promoting transparent and contextualized reporting.

Beyond its immediate use for reporting individual studies, the systematic use of the Qual-CBMA checklist could also facilitate meta-research and, in the longer term, automated reviews. The use of clearly defined criteria with explicit comments enables structured extraction of methodological features across CBMAs, facilitating large-scale analyses of reporting practices and methodological trends. In this way, Qual-CBMA may contribute not only to transparency at the CBMA level but also to cumulative methodological insight at the field level.

We hope that the Qual-CBMA checklist promotes greater methodological transparency and consistency in CBMA of neuroimaging data. By systematically verifying whether each of the 15 criteria is met, researchers will be able to identify methodological strengths and limitations early in the review or design process. The checklist encourages explicit reporting of deviations from best practices and provides a structured framework for discussing their potential implications. When applied across studies, Qual-CBMA should strengthen the overall credibility of evidence synthesis in neuroimaging. We anticipate that its adoption by authors, reviewers, and editors will help standardize the evaluation of CBMAs and foster cumulative progress in neuroimaging meta-analyses, thereby enhancing the methodological quality of the field and strengthening its contribution to neuroscience. Future updates may refine these criteria as new methodological advances and validation studies emerge.

## Data Availability

The original contributions presented in the study are included in the article/Supplementary material, further inquiries can be directed to the corresponding author.

## Footnotes

1
https://www.crd.york.ac.uk/prospero/

2
https://osf.io/
