# Supplementary material for: Evaluating the methodological quality of coordinate-based meta-analyses: the qual-CBMA checklist
Source: Front Neuroimaging. 2026 May 13;5:1753543. doi: 10.3389/fnimg.2026.1753543 (PMC13212178; doi:10.3389/fnimg.2026.1753543)
Supplement: Supplementary file 1 [file Table_1.docx]

Supplementary Material

# Exploratory search

To explore whether coordinate-based meta-analyses (CBMAs) adhere to the so-called “ten simple rules” recommendations, we conducted a systematic search at PubMed with the keywords ("voxel-based" OR "coordinate-based") AND "meta-analysis" and included all CBMAs of human neuroimaging studies published in 2019 – we considered this scope sufficient, as the purpose of this analysis was merely illustrative for the introduction of the present manuscript rather than an exhaustive review. We excluded: (a) separate CBMAs for individuals with a condition of interest (e.g., a mental disorder) and controls when the primary analysis of the manuscript was the comparison between these groups; (b) CBMAs combining several conditions of interest (e.g., multiple mental disorders) when the primary analyses were the condition-specific CBMAs; and (c) any complementary analyses (e.g., subgroup or multimodal analyses). When a manuscript reported CBMAs on multiple imaging modalities (e.g., voxel-based morphometry (VBM) and fMRI), we included all of them. When separate analyses were conducted for opposite contrasts (e.g., activations and deactivations), we treated them as a single CBMA. Two researchers (LF and AS) performed the search independently and resolved any disagreements by consensus.

We extracted the following information from each included CBMA: meta-analytic method used; whether it cited the best-practice guidelines; whether all included studies had used similar imaging modalities/measures (e.g., all included studies had acquired structural MRIs and conducted voxel-based morphometry), functional paradigms (if applicable; e.g., all included studies had used cognitive tasks), and conditions (if applicable; e.g., all included investigated the same mental disorder); the number of datasets and their sample sizes; the design (one-sample vs. two-sample); whether the paper reported search keywords and inclusion/exclusion criteria; whether it included only results in which the statistical threshold does not depend on the brain region; whether the search volumes of the included datasets and the CBMA agreed (e.g., that they examined the same brain regions); whether coordinates had been transformed into a common space (e.g., MNI or Talairach) before conducting the analyses; whether it consisted of only one GLM contrast per dataset or, if it includes two or more GLM contrasts from the same study (e.g., sad faces vs. neutral faces, and sad faces vs. fixation), it appropriately adjusted for multiple GLM contrasts; whether CBMA manuscript reported that the data retrieved from the studies’ manuscripts had been collected independently by at least two investigators; whether the protocol had been preregistered; the statistical significance level; whether it had conducted any diagnostic test (e.g., assessing potential publication bias); and whether it described the included datasets, (e.g., specifying the mean age and percentage of females of their samples). Two researchers (LF and AS) independently collected these data.

We considered that a CBMA followed the “rule” of a clear definition of the research question if all included studies addressed the same question and therefore used the same imaging modality (e.g., DTI) or measure (e.g., cortical thickness), functional paradigm, and conditions (e.g., all studies used task-based fMRI to investigate working memory in bipolar disorder). A CBMA was considered to follow the “simple rule” on power if it included at least twenty datasets, each comprising at least twelve participants (per group in two-sample designs). We acknowledge that these sample sizes are modest, but our aim was merely to identify clear departures from the guideline rather than to impose a stringent power requirement. A CBMA was judged to follow the “simple rule” on systematic search and data collection if the paper reported search keywords for at least one scientific database and specified inclusion and exclusion criteria. To evaluate compliance with the “simple rule” on consistent search coverage and reference space, we verified that: (a) all included results used statistical thresholds independent of brain region; (b) the search volumes of the included datasets and the CBMA were consistent; and (c) data were transformed into a common space before analysis. A CBMA was considered to follow the “simple rule” on adjustment for multiple GLM contrasts if it included only one contrast per dataset or applied an appropriate adjustment for multiple contrasts. We considered that a CBMA followed the “simple rule” on data checking if the manuscript reported that at least two investigators independently extracted data from the original manuscripts. Compliance with the “simple rule” on protocol preregistration was assumed when a preregistered protocol was available. We did not evaluate the “simple rule” on balancing sensitivity and susceptibility to false positives, as the significance threshold may depend on the specific goals of the CBMA (e.g., exploratory vs. confirmatory). Finally, a CBMA was judged to follow the “simple rule” on diagnostic testing if it reported any diagnostic analyses, and to follow the “simple rule” on reporting transparency if it described the included datasets, GLM contrasts, and samples.

We ultimately included 23 CBMAs that cited the best-practice guidelines and 79 CBMAs that did not. The meta-analytic methods used were Activation Likelihood Estimation (ALE) (Turkeltaub, Eden et al. 2002, Laird, Fox et al. 2005, Eickhoff, Laird et al. 2009, Eickhoff, Bzdok et al. 2012, Eickhoff, Nichols et al. 2016), Seed-based d Mapping (SDM) (Radua and Mataix-Cols 2009, Radua, Mataix-Cols et al. 2012, Radua, Rubia et al. 2014, Albajes-Eizagirre, Solanes et al. 2019, Fortea, Ortuno et al. 2025), and Multilevel Kernel Density Analysis (MKDA) (Wager, Lindquist et al. 2007).

The results showed that among the CBMAs that cited the best-practice guidelines, none of the CBMAs addressed all recommendations explicitly in their reporting, which may reflect differences in relevance, interpretation, or reporting conventions. Specifically, 43% followed at least eight recommendations, and 57% followed fewer (five to seven). All CBMAs were transparent in reporting the datasets, 96% conducted a systematic search, 96% used the same search coverage and reference space, 83% included only one GLM contrast or appropriately adjusted for multiple contrasts, and 70% included an adequate amount of data. Conversely, only 52% were specific about the research question, 52% included diagnostic tests, 43% performed duplicate data extraction, and 9% had preregistered their protocol.

CBMAs that did not cited the best-practice guidelines displayed a broadly similar profile, although they more frequently included inadequate amounts of data (70% vs. 30%; Fisher’s exact test, p = 0.001) and more often failed to use the same search coverage and reference space (39% vs. 4%; p < 0.001).

This exploratory assessment is not intended as a critique of past work, but simply highlights how reporting practices vary across studies.

# 2. Sample size simulations: Table 2

Out = NULL

for (n in 5:38) {

Out = rbind(Out, data.frame(

n,

one_samp = round(power.t.test(

type = "one.sample",

n = n,

alternative = "one.sided",

delta = 1,

sig.level = 0.001

)$power / 0.8, 2),

n,

two_samp = round(power.t.test(

type = "two.sample",

n = n,

alternative = "one.sided",

delta = 1,

sig.level = 0.001)$power / 0.8, 2),

# Second column for two-sample:

n = n + 34,

two_samp = round(power.t.test(

type = "two.sample",

n = n + 34,

alternative = "one.sided",

delta = 1,

sig.level = 0.001)$power / 0.8, 2)

))

}

print(Out)

**3. Sample size simulations – Inclusion of statistical maps**

# Simulate meta-analyses (*n* studies, with the first *n.maps* being maps)

library(metansue)

sim_nim <- function (d, n, n.maps) {

# Create random studies’ sample sizes

studies.n = sample(c(10, 15, 20, 30, 40, 50), n, replace = TRUE)

t <- as.numeric(c())

# For each study:

for (study_i in 1:n) {

# Simulate individual participant data

x = rnorm(studies.n[study_i], d)

# Conduct the t-test

ttest = t.test(x, alternative = "greater")

t_i = ttest$statistic

# If it is not statistically significant (i.e., not peak) and the

# statistical map is not available, convert to NA

if (ttest$p.value > 0.001 && study_i > n.maps) {

t_i = NA

}

t = c(t, t_i)

}

# Conduct meta-analysis with MetaNSUE

m = meta(smc_from_t(t, studies.n, alpha = 0.001))

m$hypothesis$z

}

X = NULL

for (d in c(0.2, 0.5, 0.8)) {

for (n in 10:20) {

for (n.maps in 0:n) {

cat("Simulating", n, "studies /", n.maps, "maps\n")

z = c()

for (nim in 1:100) {

z = c(z, sim_nim(d, n, n.maps))

}

X = rbind(X, data.frame(d, n, n.maps, z = mean(z)))

}

}

}

# Find the equivalent number of studies from z

X$eq.n = NA

for (d in c(0.2, 0.5, 0.8)) {

# In studies without maps, model sqrt(n) ~ z, derived from z ~ sqrt(n)

i = which(X$n.maps == 0 & X$d == d)

m = lm(sqrt(X$n[i]) ~ X$z[i])

# In all studies, estimate equivalent n from z according to the model

i = which(X$d == d)

X$eq.n[i] = (coef(m)[1] + coef(m)[2] * X$z[i])^2

}

# Model the difference between equivalent and actual number of studies

# depending on the number of maps

m = lm(X$eq.n - X$n ~ 0 + X$n.maps)

cat("Increase of", round(coef(m), 2), "per map\n")

for (d in c(0.2, 0.8)) {

i = which(X$d == d)

m = lm((X$eq.n - X$n)[i] ~ 0 + X$n.maps[i])

cat("d", d, "- Increase of", round(coef(m), 2), " per map \n")

}
